# The art of nomograms

**DOI:** 10.1186/s40662-018-0096-z

**Published:** 2018-01-25

**Authors:** Samuel Arba Mosquera, Diego de Ortueta, Shwetabh Verma

**Affiliations:** 1 Recognized Research Group in Optical Diagnostic Techniques, University of Valladolid, Valladolid, Spain; 2 SCHWIND eye-tech-solutions, Mainparkstr. 6-10, D-63801 Kleinostheim, Germany; 3 Department of Ophthalmology and Sciences of Vision, University of Oviedo, Oviedo, Spain; 4 Augenzentrum Recklinghausen, Recklinghausen, Germany; 5 Experimental Radiation Oncology, University Medical Center Mannheim, Heidelberg University, Heidelberg, Germany; 6 Interdisciplinary Center for Scientific Computing (IWR), Heidelberg University, Heidelberg, Germany; 7 Central Institute for Computer Engineering (ZITI), Heidelberg University, Heidelberg, Germany

**Keywords:** Refraction, Nomograms, Refractive outcomes, Sphere, Cylinder

## Abstract

**Background:**

To retrospectively analyse strategies for adjusting refractive surgery plans with reference to the preoperative manifest refraction.

**Methods:**

We constructed seven nomograms based on the refractive outcomes (sphere, cylinder, axis [SCA]) of 150 consecutive eyes treated with laser in situ keratomileusis for myopic astigmatism. We limited the initial data to the SCA of the manifest refraction. All nomograms were based on the strategy: if for x diopters (D) of attempted metric, y D is achieved; we can reverse this sentence and state for achieving y D of change in the metric, x D will be planned. The effects of the use of plus or minus astigmatism notation, spherical equivalent, sphere, principal meridians notation, cardinal and oblique astigmatism, and astigmatic axis were incorporated.

**Results:**

All nomograms detected subtle differences in the spherical component (*p* < 0.0001). Nomograms 5 and 7 (using power vectors) and 6 (considering axis shifts) detected significant astigmatic differences (nomogram 5, *p* < 0.001; nomogram 6, *p* < 0.05; nomogram 7, *p* < 0.005 for cardinal astigmatism, *p* = 0.1 for oblique astigmatism). We observed mild clinically relevant differences (~ 0.5 D) in sphere or astigmatism among the nomograms; differences of ~ 0.25 D in the proposals for sphere or cylinder were not uncommon. All nomograms suggested minor improvements versus actual observed outcomes, with no clinically relevant differences among them.

**Conclusions:**

All nomograms anticipated minor improvements versus actual observed outcomes without clinically relevant differences among them. The minimal uncertainties in determining the manifest refraction (~ 0.6 D) are the major limitation to improving the accuracy of refractive surgery nomograms.

## Background

Nomograms have been used from the beginning of refractive surgery. In 1998, Yang et al. [1] evaluated a commercially available neural network program for calculating photorefractive keratectomy treatment nomograms and concluded that neural networks offer a potential means of segmenting and refining treatment nomograms to account for patient demographics, preoperative examinations, surgeon style, and equipment bias.

The primary reasons for developing nomograms in refractive surgery are to avoid surgical retreatments [2] and achieve an optimum refractive result. The need for having nomograms is catalysed by variability. To achieve optimum results, the laser systems available in the markets can be optimized based on their technical specifications, but the results of refractive surgery also depend on the subtle differences among lasers, surgeons, operation room environment and the patient population and demographics. Thus, to compensate for these differences and obtain optimum results, different values are used by surgeons when compared to the actual patient data. These adjustments are determined using Nomograms. Nomograms are designed after precisely analysing a range of patient data based on several factors contributing variability. The precision and success of a nomogram rests on several factors such as valid algorithm design, accurate patient data and correct grouping of the data. In addition to this, nomograms must be constantly updated, as lasers, surgeons, and techniques change over time.

Feltham and Wolfe, who retrospectively analysed the effects of ablation size, refractive errors, patient age, and corneal curvature on the retreatments, found that the older the patient and the larger the refractive error, the greater the risk of not achieving a residual refractive error of ±0.50 diopter (D) 3 months postoperatively and that corneas that are steeper preoperatively have a greater risk of retreatment.

Most reported nomograms have provided accurate results for myopic spheres; however, for hyperopic spheres and astigmatic outcomes the results are less predictable. Moniz and Fernandes [3] analysed a nomogram for treating astigmatism with laser in situ keratomileusis (LASIK). Similarly, Alpins and Goggin [4] provided a method to analyse refractive outcomes of astigmatic refractive surgery. The Alpins methodology uses three principal vectors and the various ratios among them provide an aggregate analysis of astigmatic change with parallel indices for spherical correction. A comparative analysis using arithmetic and vectorial means and necessary nomogram adjustments for refining spherical and astigmatic treatments can also be derived. These advanced techniques, together with their suitability for statistical analysis, comprehensively address the outcome analysis requirements of the entire cornea and the refractive correction for examining successful refractive surgery outcomes.

However, nomograms are only useful if the effects of their proposed surgical plans can be positively verified. Gailitis [5] compared the outcomes between two different excimer laser platforms using optimized nomograms and reported good results for both platforms, but superior results were obtained with the platform using the more recent nomogram.

Nomograms can consider all kinds of variables for analysis. A recent nomogram proposed the coupling effects between preoperative high-order aberrations and refractive outcomes [6]. The authors found that patient satisfaction was slightly higher than that of patients who underwent previous laser refractive surgery at the same clinic and concluded that the advanced nomogram increased treatment accuracy regarding the uncorrected visual acuity (UDVA) and the mean postoperative refraction and reduced the rate of hyperopic overcorrection compared with earlier studies. The need for retreatment procedures decreased, and patient satisfaction was high.

There is no single approach for adjusting the surgical parameters based on retrospective analyses of previous outcomes. Arnalich-Montiel et al. [7] examined four systematic strategies and one intuitive approach for adjusting the ablation sphere in myopic wavefront LASIK with reference to preoperative manifest refraction. Surprisingly, they found that the postoperative manifest refraction spherical equivalent varied lesser when non-systematic, intuitive adjustments to the ablation sphere were used. There was a strong trend toward reduced variability in the results in patients with a larger wavefront diameter. The authors concluded that back-calculation to model results with different pre-treatment ablation adjustment strategies might be useful to eliminate unpromising new approaches before clinical trials.

A nomogram can be designed based on several factors. One can analyse the factors as variables that are used to design an equation that describes an empirical data precisely. Looking at this process purely mathematically, the more variables that are included in the analysis the better the fit becomes. However, there is no ideal criterion to judge the relevance of the analysed factors on the refractive outcomes. These factors are selected mostly based on scientific studies, common sense and sometimes even a feeling. Although before including other factors, reaching an optimum basis for the refractive surgery design is imperative. In the current study, we aim at analysing the basic criteria that can be used to develop a nomogram, the Sphere Cylinder and axis (SCA) component of the manifest refraction. We analyse seven systematic strategies for adjusting the surgical plan in refractive surgery with reference to the preoperative manifest refraction (SCA), with an aim to find an optimum fundamental nomogram that can be further optimized with several other popular factors affecting refractive outcomes.

## Methods

One hundred and fifty consecutive eyes of 75 patients that had been treated with the Amaris (SCHWIND eye-tech-solutions, Kleinostheim, Germany) “aberration neutral” (Aberration-Free™) aspheric ablation profiles were analysed retrospectively.

The inclusion criteria were a bilateral surgery on the same day targeted for emmetropia, preoperative bilateral corrected distance visual acuity (CDVA) ≥ 20/25 (logarithm of the minimum angle of resolution [logMAR] ≤ 0.1), and no signs of amblyopia.

The 6-month follow-up data were available for all 150 eyes (100%). The mean preoperative manifest defocus refraction was − 3.60 ± 1.54 diopters (D) (range, − 7.50 to − 1.25 D) and the mean preoperative manifest astigmatism was 0.79 ± 0.74 D (range, 0.00 to 4.00 D). We measured the corneal topography [8] for all eyes and obtained the corneal wavefront aberrations [9, 10] up to the 7th Zernike order (36 terms) (Keratron-Scout, OPTIKON2000, Rome, Italy), manifest refraction, UDVA, and CDVA. The measurements were performed preoperatively and at 1, 3, and 6 months postoperatively.

All ablations were non-wavefront-guided but were based on aspheric [11] aberration-neutral profiles (and not on the profiles proposed by Munnerlyn [12]) to balance the induction of spherical aberration [13, 14] (prolateness optimization [15]). This approach included a multidynamic aspheric transition zone, aberration and focus shift compensation due to ablation, pseudomatrix-based spot positioning, and enhanced compensation for the loss of efficiency [16]; all were based on theoretical equations validated with ablation models and clinical evaluations.

We used a 6.3-mm central, fully corrected optical zone (OZ) for myopia and a 7.0-mm OZ for high astigmatism, together with a variable transition size that was provided automatically by the laser depending on the planned refractive correction (range, 6.5–9.2 mm). The ablation was performed using the AMARIS excimer laser (SCHWIND eye-tech-solutions, Kleinostheim, Germany), which is a flying-spot laser system that uses a real ablative spot volume locally considered through a self-constructing algorithm that controls for the local repetition rates to minimize the thermal load of the treatment [17].

The AMARIS laser system works at a repetition rate of 750 Hz and produces a beam (size, 0.54 mm) (full-width-at-half-maximum) and a super-Gaussian spot profile [18, 19]. High-speed eye tracking (pupil and limbus tracker with cyclotorsional tracking [20]) with a 1050 Hz acquisition rate is accomplished with a 3-ms latency time [21].

We based our nomogram analyses on three manifest refraction values (sphere, cylinder, axis [SCA]), and for simplicity we ignored other known factors of refractive deviation such as treatment duration [22], in that increased dehydration leads to different laser tissue interaction attributes; OZ [23–26], in that larger zones provide more natural corneal shapes but smaller zones save ablated tissue; age [2, 24], in that corneal water content and accommodation decrease with age; treated eye [27, 28], in the sense of bilateral symmetry for astigmatism; keratometric values [2, 27], in that greater loss of efficiency affects ablation of steeper corneas; wavefront refraction [7, 29–31]; coupling effects [28]; effects of wave aberration on the manifest refraction [24, 32]; and neural adaptation [25, 33].

For all nomogram construction, we used linear correlation as a nomogram proposal: if for x D of attempted metric, y D is achieved; we can reverse this sentence and state that for achieving y D of change in the metric, x D will be planned.

### Nomogram 1: sphere and negative cylinder

Spherocylindrical (SCA) prescriptions can be easily converted to a plus or minus astigmatism notation.

1 $$ {S}_{conv}={S}_{orig}+{C}_{orig} $$

2 $$ {C}_{conv}=-{C}_{orig} $$

3 $$ {A}_{conv}=\operatorname{mod}\left({A}_{orig}+90,180\right) $$

where conv denotes the converted SCA prescription, and orig denotes the original SCA prescription.

In this approach, we correlated the achieved spherical change (considering astigmatism as the negative convention) with the attempted change and the magnitude of the achieved cylindrical change (vectorial analysis with astigmatism as the negative convention) with the attempted change (all at the corneal plane where the ablation is performed).

### Nomogram 2: sphere and positive cylinder

We correlated the achieved spherical change (considering astigmatism as the positive convention) with the attempted change and the magnitude of the achieved cylindrical change (vectorial analysis with astigmatism as the positive convention) with the attempted change (all at the corneal plane where the ablation is performed).

### Nomogram 3: spherical equivalent and astigmatism

We correlated the achieved spherical equivalent change with the attempted change, and the absolute magnitude of the achieved cylindrical change (vectorial analysis) with the attempted change (all at the corneal plane where the ablation is performed).

### Nomogram 4: principal meridians

Spherocylindrical (SCA) prescriptions can be easily converted to the principal meridians notation.

4 $$ {Cyl}_1={S}_{orig} $$

5 $$ {Cyl}_2={S}_{orig}+{C}_{orig} $$

where Cyl_1_ and Cyl_2_ are the meridional powers of the principal meridians. We correlated the achieved meridional power change (vectorial analysis) with the attempted power change for the principal meridians at the corneal plane where the ablation is performed.

### Nomogram 5: spherical equivalent and cardinal and oblique astigmatism

Spherocylindrical (SCA) prescriptions can be converted easily to power vector notation of the form [C_+_, M, C_x_].

6 $$ {C}_{+}=-\frac{C}{2}\cos (2A) $$

7 $$ M=S+\frac{C}{2} $$

8 $$ {C}_{\times }=-\frac{C}{2}\sin (2A) $$

where M is the spherical equivalent (defocus component), C_+_ the cardinal astigmatism, and C_x_ the oblique astigmatism. The three components, respectively, represent the power of a Jackson crossed-cylinder with axes at 0 degree and 90 degrees, the spherical equivalent power, and the power of a Jackson crossed-cylinder with axes at 45 degrees and 135 degrees. We correlated the achieved spherical equivalent change with the attempted change, and the achieved cylindrical change (cardinal and oblique components analysis) with the attempted change (all at the corneal plane where the ablation is performed).

### Nomogram 6: spherical equivalent and astigmatism considering residual cyclotorsion

We correlated the achieved spherical equivalent change with the attempted change, and the achieved cylindrical change (cardinal and oblique components analysis) with the attempted change (all at the corneal plane where the ablation is performed). Moreover, for astigmatism we compared the achieved astigmatic axis to the attempted axis.

Assuming that the freedom to adjust the applied magnitudes of astigmatism exists, an optimal factor that minimizes the magnitude of the residual astigmatism was calculated:

9 $$ \left|{Cyl}_{Post}\right|=\left|{Cyl}_{Pre}\right|\sqrt{F^2-2F\cos \left(2\theta \right)+1} $$

where F is the adjustment factor due to cyclotorsion and θ is the residual cyclotorsion.

The factor that minimizes residual astigmatism is the one that minimizes the square root in the formula above, thus, the factor that minimizes the quadratic function is inside the square root.

The minimum of this function is the vertex of the parabola:

10 $$ F=\cos \left(2\theta \right) $$

In addition, the residual astigmatic magnitude is:

11 $$ \left|{Cyl}_{Post}\right|=\left|{Cyl}_{Pre}\right|\sin \left(2\theta \right) $$

Notice that F is always between [− 1, + 1] and that F changes its sign when:

12 $$ \theta =\frac{\pi }{4} $$

As π/4 is much larger than typical cyclotorsion errors, it can be assumed that for practical purposes F is always positive.

### Nomogram 7: power vectors analysis

We correlated the achieved spherical equivalent change to the attempted change, and the achieved cylindrical change (separate cardinal and oblique components analyses) to the attempted change (all at the corneal plane where the ablation is performed).

### Statistical analyses

We assessed the statistical significance of the nomogram correction compared to the preoperative refraction using paired Student’s t-tests.

We assessed the statistical significance of the correlations using the Student’s t-tests. The coefficient of determination (r^2^) was used, and the significance of the correlations has been evaluated considering a metric distributed approximately as t with N-2 degrees of freedom where N is the size of the sample.

The level of statistical significance was *p* < 0.05.

## Results

### Refractive outcomes

At 6 months postoperatively, the spherical equivalent and the cylinder decreased significantly to subclinical levels: the mean residual defocus refraction was − 0.08 ± 0.36 D (range, − 1.12 to + 0.75 D; *p* < 0.0001) and mean residual astigmatism 0.16 ± 0.21 D (range, 0.00 to 0.75 D; *p* < 0.001). In 85% of eyes (*n* = 127), the spherical equivalent was within ±0.50 D of emmetropia, and in 97% of eyes (*n* = 145), the astigmatism was within ±0.50 D of emmetropia (Table 1).

**Table 1:** Comparison of refractive outcomes 6 months after surgery for all 150 eyes

	Pre-op (Mean ± SD)	Post-op (Mean ± SD)	*P* value
Spherical equivalent (D)	−3.60 ± 1.54	− 0.08 ± 0.36	< 0.0001*
Cylinder (D)	0.79 ± 0.74	0.16 ± 0.21	< 0.001*
Predictability within ±0.50 D (%)	–	85% for Seq 97% for Ast	–
Predictability within ±1.00 D (%)	–	97% for Seq 100% for Ast	–

*Ast* = astigmatism; *Seq* = spherical equivalent

*Statistically significant

### Nomogram 1: sphere and negative cylinder

**Fig. 1:**
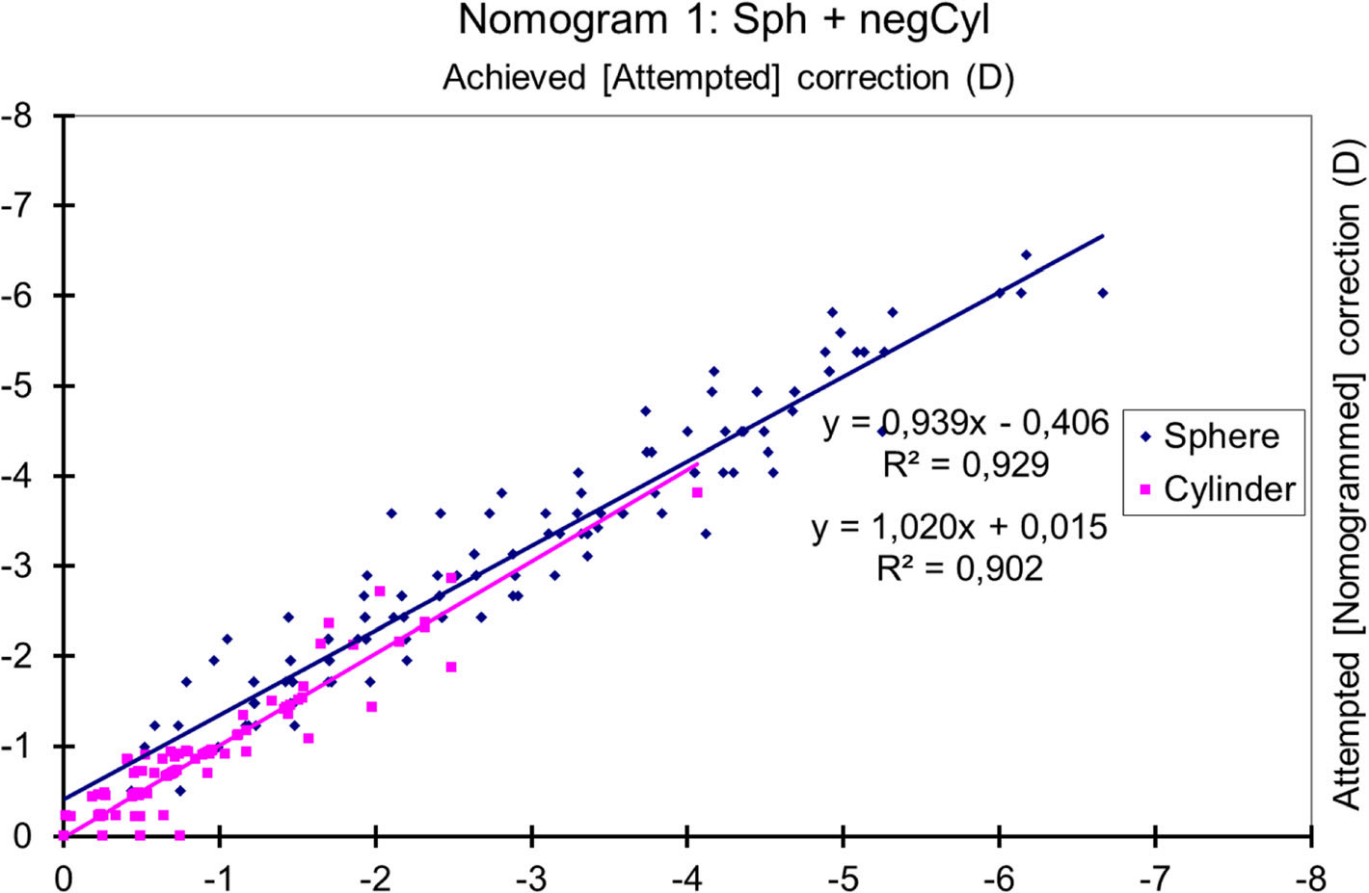
Achieved correction vs. Attempted correction with Nomogram 1. The achieved spherical change (considering astigmatism as a negative convention) correlated with the attempted change (*p* < 0.0001), and the magnitude of the achieved cylindrical change (vectorial analysis with astigmatism as the negative convention) correlated with the attempted change (*p* < 0.0001). The nomogram correction differs significantly from the preoperative refraction for sphere (*p* < 0.0001) but not for astigmatism (*p* = 0.3). The nomogram proposes a − 6% reduction in sphere with a − 0.4 D bias, and a + 1% enhancement of the cylinder. A refraction of − 2.50 D sphere with − 1.50 D astigmatism at 63 degrees should be planned as − 2.77 D sphere with − 1.52 D astigmatism at 63 degrees

The achieved spherical change (considering astigmatism as a negative convention) was correlated with the attempted change (*p* < 0.0001), and the magnitude of the achieved cylindrical change (vectorial analysis with astigmatism as a negative convention) was correlated with the attempted one (*p* < 0.0001) (Fig. 1).

The nomogram correction differed significantly from the preoperative refraction for sphere (*p* < 0.0001) with a mean nomogram correction of − 0.23 ± 0.39 D (range, − 1.49 D to + 0.76 D; median − 0.25 D) but not for astigmatism (*p* = 0.3).

### Nomogram 2: sphere and positive cylinder

**Fig. 2:**
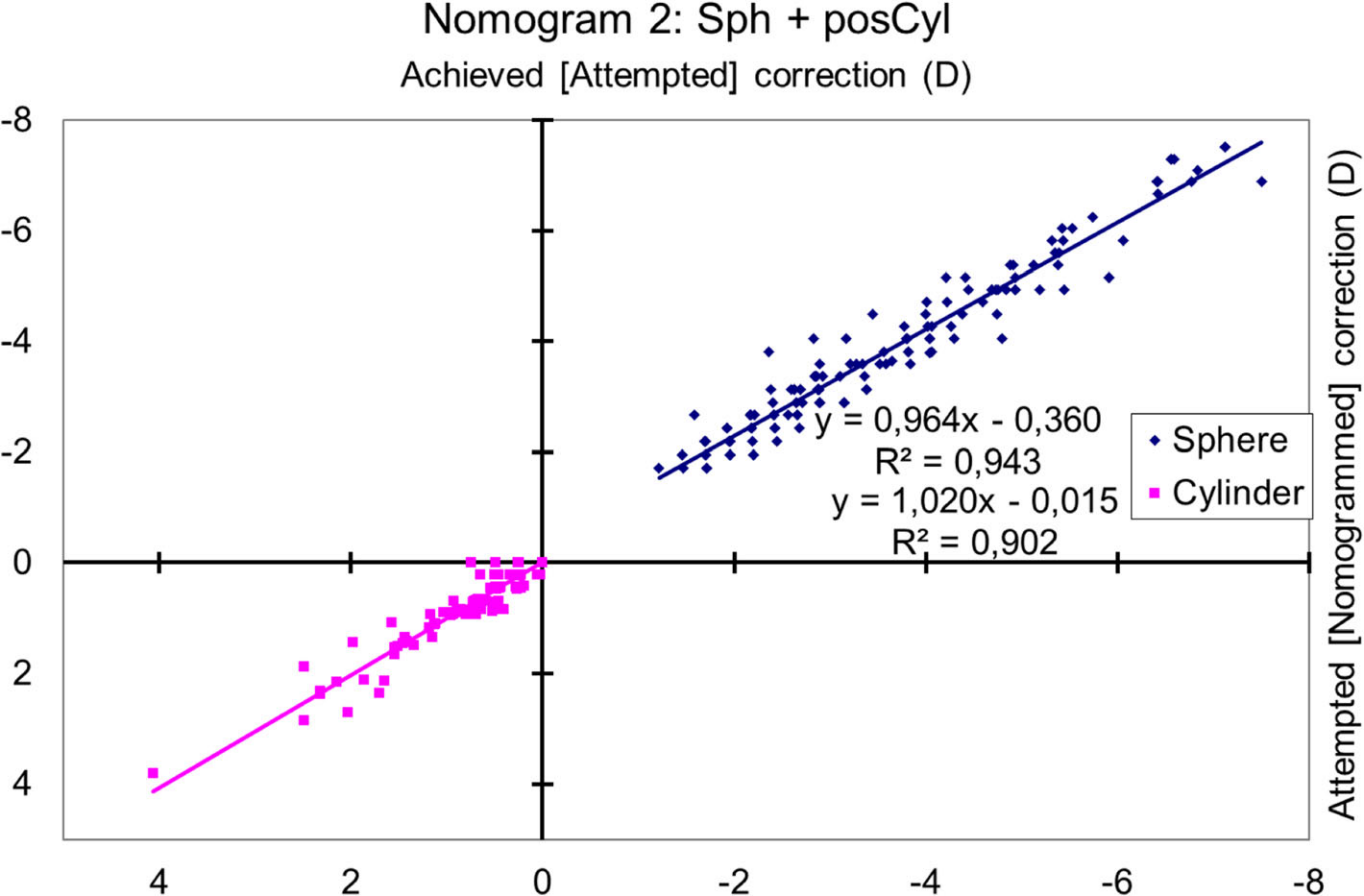
Achieved correction vs. Attempted correction with Nomogram 2. The achieved spherical change (considering astigmatism as a positive convention) correlated with the attempted change (*p* < 0.0001), and the magnitude of the achieved cylindrical change (vectorial analysis with astigmatism as a positive convention) with the attempted change (*p* < .0001). The nomogram correction differs significantly from the preoperative refraction for sphere (*p* < 0.0001) but not for astigmatism (*p* = 0.3). The nomogram proposes a − 4% reduction in sphere with a − 0.39 D bias and a + 1% enhancement of the cylinder. A refraction of − 4.00 D sphere with + 1.50 D astigmatism at 153 degrees (− 2.50 D sphere with − 1.50 D astigmatism at 63 degrees) should be planned as − 4.25 D sphere with + 1.52 D astigmatism at 153 degrees (− 2.73 D sphere with − 1.52 D astigmatism at 63 degrees)

The achieved spherical change (considering astigmatism as a positive convention) was correlated with the attempted correction (*p* < 0.0001), and the magnitude of the achieved cylindrical change (vectorial analysis with astigmatism as a positive convention) was correlated with the attempted change (*p* < 0.0001) (Fig. 2).

The nomogram correction differed significantly from the preoperative refraction for sphere (*p* < 0.0001) with a mean nomogram correction of − 0.23 ± 0.36 D (range, − 1.46 D to + 0.76 D; median − 0.25 D) but not for astigmatism (*p* = 0.3).

### Nomogram 3: spherical equivalent and astigmatism

**Fig. 3:**
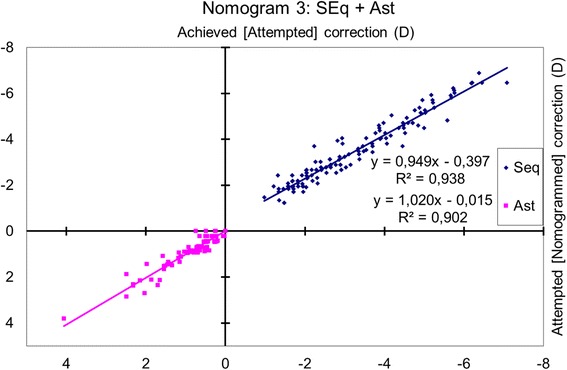
Achieved correction vs. Attempted correction with Nomogram 3. The achieved spherical equivalent change correlated with the attempted change (*p* < 0.0001), and the absolute magnitude of the achieved cylindrical change (vectorial analysis) with the attempted change (*p* < 0.0001). The nomogram correction differs significantly from the preoperative refraction for sphere (*p* < 0.0001) but not for astigmatism (*p* = 0.3). The nomogram proposes a − 5% reduction in sphere with a − 0.41 D bias, and a + 1% enhancement of the cylinder. A refraction of − 2.50 D sphere with − 1.50 D astigmatism at 63 degrees should be planned as − 2.75 D sphere with − 1.52 D astigmatism at 63 degrees

The achieved spherical equivalent change was correlated with the attempted change (*p* < 0.0001), and the absolute magnitude of the achieved cylindrical change (vectorial analysis) was correlated with the attempted change (*p* < 0.0001) (Fig. 3).

The nomogram correction differed significantly from the preoperative refraction for sphere (*p* < 0.0001), with a mean nomogram correction of − 0.23 ± 0.36 D (range, − 1.47 D to + 0.76 D; median − 0.25 D) but not for astigmatism (*p* = 0.3).

### Nomogram 4: principal meridians

**Fig. 4:**
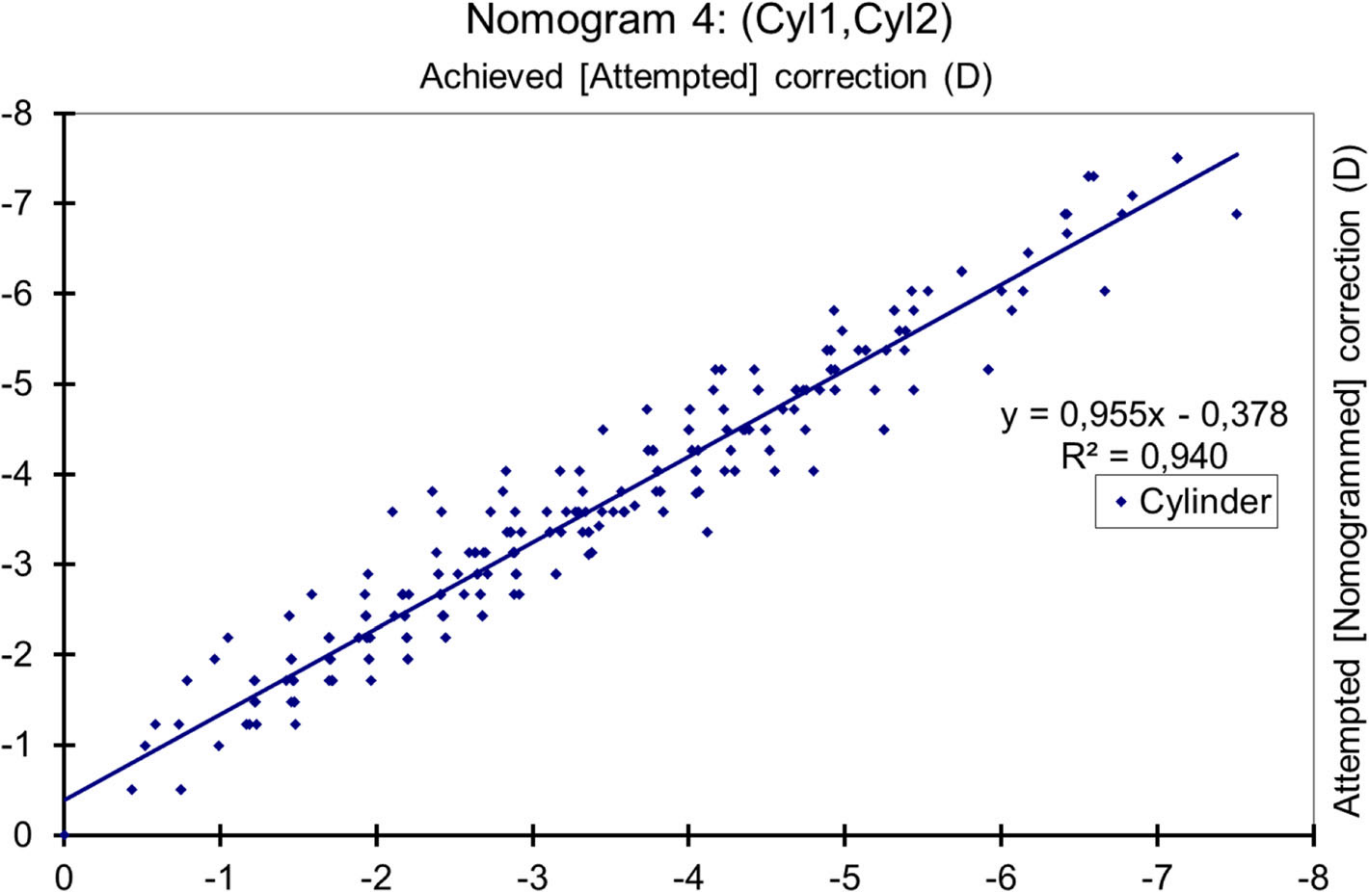
Achieved correction vs. Attempted correction with Nomogram 4. The achieved meridional power change correlated with the attempted change (*p* < 0.0001) and the nomogram correction differs significantly from the preoperative refraction (*p* < 0.0001). The nomogram proposes a − 5% reduction in planned meridional power with a − 0.39 D bias. A refraction of − 2.50 D sphere with − 1.50 D astigmatism at 63 degrees should be planned as − 2.79 D sphere with − 1.44 D astigmatism at 63 degrees

The achieved meridional power change at the principal meridians was correlated with the attempted power change (*p* < 0.0001) (Fig. 4), and the nomogram correction differed significantly from the preoperative refraction (*p* < 0.0001), with a mean nomogram correction of − 0.23 ± 0.37 D (range, − 1.49 D to + 0.76 D; median − 0.25 D).

### Nomogram 5: spherical equivalent and cardinal and oblique astigmatism

**Fig. 5:**
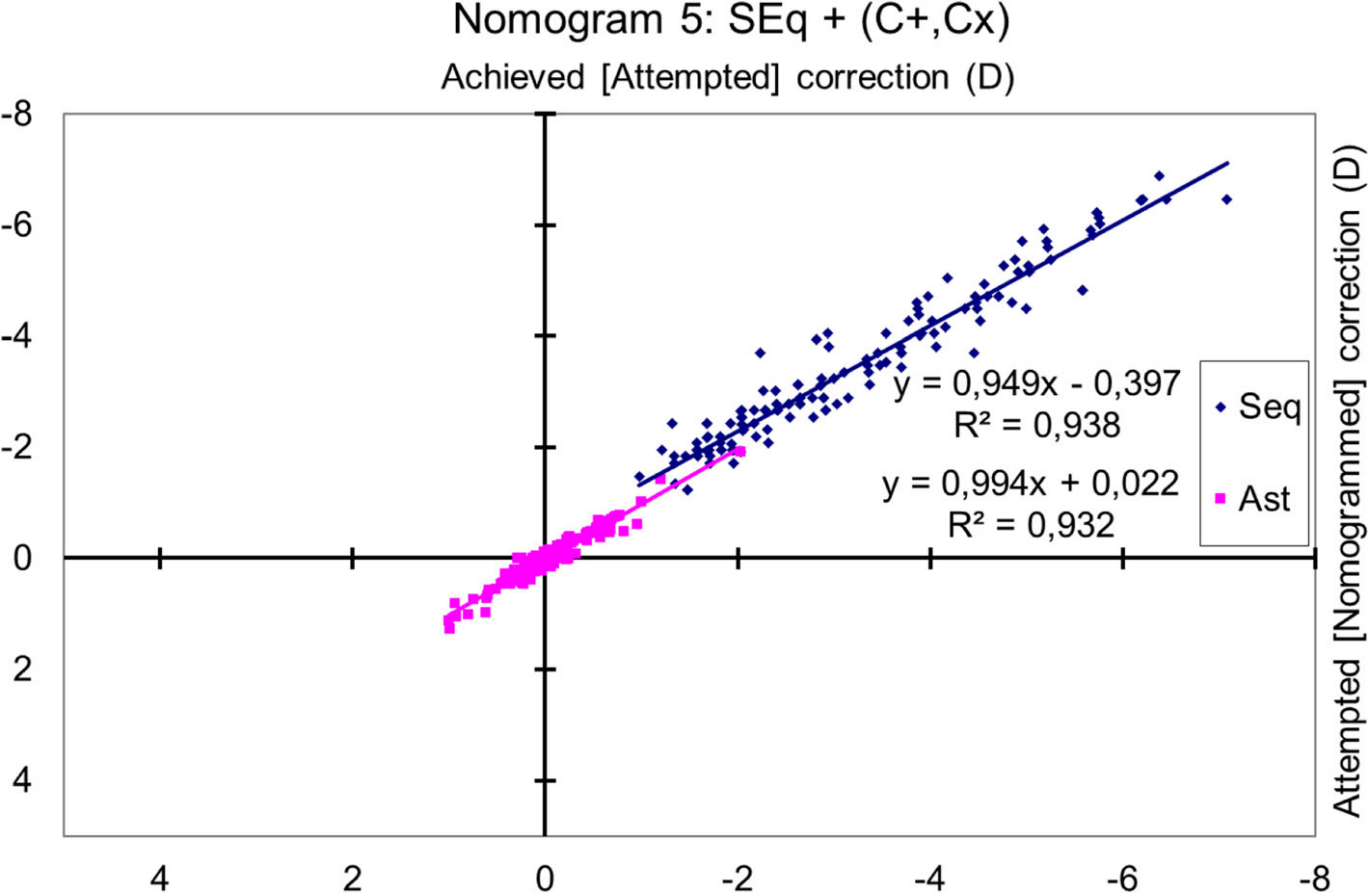
Achieved correction vs. Attempted correction with Nomogram 5. The achieved spherical equivalent change correlated with the attempted change (*p* < 0.0001), and the absolute magnitude of the achieved cylindrical change (cardinal and oblique components analysis) with the attempted change (*p* < 0.0001). The nomogram correction differs significantly from the preoperative refraction for sphere (*p* < 0.0001) and for astigmatism (*p* < 0.001). The nomogram proposes a − 5% reduction in sphere with a − 0.41 D bias, and a − 1% reduction of the cylinder with a + 0.01 D bias. A refraction of − 2.50 D sphere with − 1.50 D astigmatism at 63 degrees should be planned as − 2.75 D sphere with − 1.52 D astigmatism at 63 degrees

The achieved change in the spherical equivalent was correlated with the attempted change (*p* < 0.0001), and the achieved cylindrical change (cardinal and oblique components analysis) was correlated with the attempted change (*p* < 0.0001) (Fig. 5).

The nomogram correction differed significantly from the preoperative refraction for sphere (*p* < 0.0001), with a mean nomogram correction of − 0.23 ± 0.36 D (range, − 1.47 D to + 0.76 D; median, − 0.25 D) and for astigmatism (*p* < 0.001), with a mean nomogram correction of + 0.02 ± 0.09 D (range, − 0.29 D to + 0.37 D; median, 0.00 D).

### Nomogram 6: spherical equivalent and astigmatism considering residual cyclotorsion

The achieved spherical equivalent change was correlated with the attempted change (*p* < 0.0001), and the absolute magnitude of the achieved cylindrical change (vectorial analysis) was correlated with the attempted change (*p* < 0.0001) (Fig. 3).

Comparing the achieved astigmatic axis to the attempted astigmatic axis, we detected a mean cyclotorsion of 6 degrees that corresponded to a compensatory factor for the astigmatism of:

13 $$ F=0.9806 $$

The nomogram correction differed significantly from the preoperative refraction for sphere (*p* < 0.0001) with a mean nomogram correction of − 0.23 ± 0.36 D (range, − 1.47 D to + 0.76 D; median − 0.25 D) and for astigmatism (*p* < 0.05), with a mean nomogram correction of − 0.02 ± 0.21 D (range, − 0.75 D to + 0.66 D; median 0.00 D).

A refraction of − 2.50 D sphere with − 1.50 D of astigmatism at 63 degrees should be planned as − 2.76 D sphere with − 1.49 D astigmatism at 63 degrees.

### Nomogram 7: power vectors analysis

**Fig. 6:**
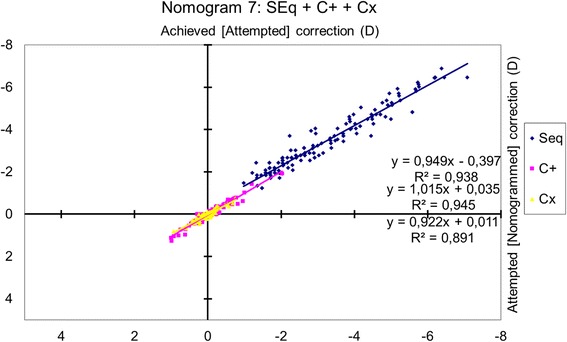
Achieved correction vs. Attempted correction with Nomogram 7. The achieved spherical equivalent change correlated with the attempted change (*p* < 0.0001) and the absolute magnitude of the achieved cylindrical change (separate cardinal and oblique components analyses) with the attempted change (*p* < 0.0001 for cardinal astigmatism; *p* < 0.0001 for oblique astigmatism). The nomogram correction differs significantly from the preoperative refraction for sphere (*p* < 0.0001) and for cardinal astigmatism (*p* < 0.005) but not for oblique astigmatism (*p* = 0.1). The nomogram proposes a − 5% reduction in sphere with a − 0.41 D bias, a + 1% enhancement of the cardinal astigmatism with a + 0.03 D bias, and a − 8% reduction of the oblique astigmatism with a + 0.01 D bias. A refraction of − 2.50 D sphere with − 1.50 D astigmatism at 63 degrees should be planned as − 2.78 D sphere with − 1.47 D astigmatism at 66 degrees

The achieved spherical equivalent change was correlated with the attempted change (*p* < 0.0001), and the achieved cylindrical change (separate cardinal and oblique components analyses) was correlated with the attempted change (*p* < 0.0001 for cardinal astigmatism; *p* < 0.0001 for oblique astigmatism) (Fig. 6).

The nomogram correction differed significantly from the preoperative refraction for sphere (*p* < 0.0001) with a mean nomogram correction of − 0.23 ± 0.36 D (range, − 1.47 D to + 0.76 D; median − 0.25 D) and for cardinal astigmatism (*p* < 0.005) with a mean nomogram correction of + 0.03 ± 0.10 D (range, − 0.29 D to + 0.37 D; median 0.00 D) but not for oblique astigmatism (*p* = 0.1).

### Comparison of the different nomogram proposals

Table 2 presents some values for comparing the different nomogram proposals.

**Table 2:** Comparison of the different nomogram proposals

Attempted			Nom 1: Sph + negCyl			Nom 2: Sph + posCyl			Nom 3: SEq + Ast			Nom 4: Cyl_1_ + Cyl_2_			Nom 5: SEq + (C_+_,C_x_)			Nom 6: Cyclotorsion			Nom 7: SEq + C_+_ + C_x_			Max. internomogram difference		
Sphere (D)	Cylinder (D)	Axis (deg)	Sphere (D)	Cylinder (D)	Axis (deg)	Sphere (D)	Cylinder (D)	Axis (deg)	Sphere (D)	Cylinder (D)	Axis (deg)	Sphere (D)	Cylinder (D)	Axis (deg)	Sphere (D)	Cylinder (D)	Axis (deg)	Sphere (D)	Cylinder (D)	Axis (deg)	Sphere (D)	Cylinder (D)	Axis (deg)	Sphere (D)	Cylinder (D)	Axis (deg)
−3.00	0.00	0	− 3.25	0.02	0	−3.29	0.02	0	−3.28	0.02	0	−3.27	0.00	0	−3.26	−0.02	0	−3.28	0.02	0	−3.23	− 0.08	98	0.06	0.09	98
0.00	−2.00	15	−0.41	−2.05	15	−0.27	− 2.04	15	− 0.34	− 2.04	15	− 0.38	− 1.93	15	− 0.34	− 2.03	15	−0.35	− 2.01	15	− 0.39	− 1.93	14	0.14	0.12	1
−3.00	− 2.00	150	− 3.25	− 2.04	150	− 3.18	− 2.03	150	−3.21	− 2.03	150	− 3.27	− 1.92	150	− 3.21	− 2.02	150	−3.22	−2.00	150	−3.28	− 1.88	150	0.10	0.15	0
−1.50	−1.00	45	−1.83	− 1.01	45	− 1.78	−1.01	45	−1.81	− 1.01	45	− 1.82	−0.96	45	− 1.80	−1.02	45	−1.82	− 1.00	45	− 1.86	− 0.91	47	0.08	0.11	2
−4.75	−3.25	120	−4.90	−3.31	120	−4.82	−3.30	120	−4.84	−3.31	120	−4.95	−3.11	120	−4.86	−3.26	120	−4.86	−3.26	120	−4.91	−3.15	118	0.13	0.20	2
4.75	−3.25	75	4.11	−3.36	75	4.43	−3.33	75	4.27	−3.35	75	4.21	−3.14	75	4.25	−3.29	75	4.25	−3.30	75	4.26	−3.32	77	0.32	0.22	2
2.00	−2.00	90	1.49	−2.05	90	1.68	−2.04	90	1.58	−2.05	90	1.55	−1.93	90	1.57	−2.03	90	1.57	−2.02	90	1.62	−2.12	90	0.19	0.19	0
3.00	−6.00	165	2.44	−6.20	165	2.86	−6.14	165	2.67	−6.17	165	2.52	−5.78	165	2.62	−6.04	165	2.63	−6.08	165	2.58	−5.96	166	0.41	0.42	1

*Ast* = astigmatism; *C*_*+*_ = cardinal astigmatism; *C*_*x*_ = oblique astigmatism; *deg.* = degree; *negCyl* = negative cylinder; *Nom* = nomogram; *SEq* = spherical equivalent; *Sph* = sphere

### Adverse events

No adverse events or complications were observed intraoperatively or postoperatively. No patient needed or requested retreatment of either eye.

## Discussion

In the current study, we used non-customized aberration-neutral profiles, i.e., the ablations were optimized to induce no change in wavefront aberration within the OZ other than sphere and cylinder components, leaving all existing higher order aberrations (HOAs) unchanged because the CDVA was unaffected by the pre-existing aberrations [25]. Thus, to compensate for the induced aberrations observed with other types of profile definitions [26], several sources of aberration might be considered; some of those sources of aberration are related to the loss of efficiency of the laser ablation for non-normal incidence [34–40].

The aim of the current study was to evaluate the differences among seven systematic strategies for adjusting the surgical refractive plan in reference to the preoperative manifest refraction, based on retrospective analysis of 150 eyes treated with the AMARIS system that used an Aberration-Free ablation profile. The advantage of the Aberration-Free ablation profile is that it aims to be neutral for HOAs, leaving the visual print of the patient as it was preoperatively with the best spectacle correction.

All patients were followed for 6 months postoperatively, and, although no nomogram adjustments were applied, no patient required retreatment. A study with a longer follow-up and more eyes would provide more proof of stable outcomes, even though the refractive spherical and astigmatic results were stable after 3 months.

The average residual defocus was about − 0.1 D and the residual cylinder about 0.2 D, with 85% of the eyes within 0.50 D and 97% within 1.0 D of emmetropia. This might be specific to the SCHWIND laser, which is calibrated for all treatments to achieve − 0.2 D of residual myopia.

Although this small series of treated eyes does not allow for definitive conclusions to be drawn or evidence-based statements, our preliminary results are promising. We compared seven different approaches for constructing a nomogram for planned refraction for laser refractive surgery. For all approaches, we limited the initial data to the SCA values of the manifest refraction. All nomogram proposals were derived based on the strategy: if for x D of attempted metric, y D is achieved; we can reverse this sentence and state that for achieving y D of change in the metric, x D will be planned.

Six nomograms considered only the SC values independent of the astigmatism axis, and nomogram 7 also considered the astigmatism axis.

Nomograms 1 and 2 were identical except for the fact that they used opposite cylinder conventions (negative versus positive). These approaches have the disadvantage of depending on the astigmatic sign, which provide different results.

Nomogram 3 is the most established method for analysing refractive outcomes, and we think that together with the intuitive approach, nomogram 3 is the most extended approach for constructing nomograms for laser refractive surgery. This approach is independent of the cylinder convention, but is based on enhancement of the spherical equivalent and astigmatism regression lines without considering the astigmatic axis. The problem is that this approach considers a perfect result as that in which the attempted correction is − 1 D cylinder at 0 degrees, and the achieved correction is − 1 D cylinder at 90 degrees. That means that it may fail for datasets with only low astigmatic values, small datasets, or datasets in which large rotations of the astigmatic angle are achieved. Nomogram 6 is a modification of nomogram 3, which further considers the effects of cyclotorsion on residual astigmatism [28], i.e., it targets reducing postoperative residual astigmatism instead of enhancing the achieved astigmatism change.

Nomogram 4 uses the astigmatism value only indirectly, since it is based on correlation of the changes in the meridional optical powers. This approach is independent from the cylinder convention, but it is based on the enhancement of the meridional regression line without considering the astigmatic axis. It can be useful for small datasets since the statistical power is enhanced using only one correlation with 2 N points, instead of two correlations with N points each.

Nomograms 5 and 7 use SCA values expressed as power vectors, whereas in nomogram 5 the astigmatism nomogram is based on analysis of the cardinal and oblique astigmatism components together, and in nomogram 7 the astigmatism nomogram is based on separate analyses of the cardinal and oblique astigmatism components. These approaches are independent of the used cylinder convention and based on enhancement of the regression lines considering the astigmatic axis.

Only nomograms 5 and 7, which used power vectors, and nomogram 6, which considered the effects of cyclotorsion on residual astigmatism [28], detected significant differences for astigmatism (nomogram 5, *p* < 0.001; nomogram 6, *p* < 0.05; and nomogram 7, *p* < 0.005 for cardinal astigmatism, but *p* = 0.1 for oblique astigmatism). This means that only by considering SCA, with emphasis on the axis, subtle astigmatic differences can be detected.

Comparing the different nomogram proposals, we observed mild significant differences of almost 0.5 D in sphere or astigmatism among the different nomogram proposals; differences of about 0.25 D in the proposals for sphere or cylinder were not uncommon.

Ditzen et al. [41] compared the correction values entered into the laser with the achieved change in refraction for these eyes and incorporated the effect of OZ size and patient age. Scatterplots comparing the laser settings to achieved postoperative refractions showed a clear 20% trend toward overcorrection. This trend increased with patient age and OZ diameter.

Sheludchenko and Fadeykina [42] studied the results of excimer laser correction for mixed astigmatism using bitoric nomograms for cylinder and standard monotoric procedures and found that bitoric ablations for astigmatic corrections resulted in fewer retreatments compared to standard monotoric procedures.

Anderson et al. [43] analysed the attempted versus achieved changes in refraction. Factors such as age, corneal thickness (pachymetry), preoperative spherical equivalent refraction, preoperative cylinder, and OZ were studied to evaluate their roles in predicting the refractive outcome 6 months after LASIK. The preoperative spherical equivalent refraction and OZ size were strong predictors of the 6-month refractive outcomes, that is, with the preoperative spherical equivalent refraction and OZ size, the postoperative refraction of the patient could be strongly estimated. However, there was no correlation between the postoperative refraction and the age, preoperative cylinder, or surgeon.

Caster et al. [44] evaluated the refractive results of conventional (non-wavefront) LASIK for treating myopia and myopic astigmatism using the Alcon LADARVision 4000 excimer laser system and nomogram adjustment techniques. They found that the outcomes improved substantially throughout the development of an accurate nomogram because of continually updated regression analysis of previous refractive results.

Zaldivar et al. [45] compared five nomogram refinements accounting for accommodation, use of a 7.0-mm OZ and a 9.5-mm transition zone, a targeted mean flap diameter of 10.5-mm, sequential interruption of the laser ablation, and cleaning of the interface. They concluded that the outcomes support the observation that five surgical and technical modifications of the hyperopic LASIK procedure resulted in excellent visual quality and refractive outcomes and a low regression rate.

Mrochen et al. [46] stated that nomograms are efficient tools for improving the predictability of refractive procedures by using statistical methods to analyse the preoperative and postoperative refractive data. The authors found that using individual nomograms significantly improved the predictability of the refractive outcome. However, theoretical investigation showed that homogeneous data distribution within cohorts is a key factor for predictable nomogram calculations. Nomograms are helpful for improving refractive outcomes. However, they are currently limited to about 90% within ±0.5 D of emmetropia.

The limitations of the current study included short follow-up and no clinical study groups for which the proposed nomogram adjustments have been planned. Long-term follow-up of these eyes will help determine the stability of these results.

Refined models to analyse the refractive outcomes and derivation of adjusted nomograms may increase the accuracy of the results; however, the high validation cost and the analysis of sufficiently large datasets to convey sufficient statistical power must be examined together with the potential benefits of nomograms.

Artificial intelligence applied to nomograms may also contribute to auto-updating of the proposals for the surgical plans, e.g., weighting the refractive outcomes based on the time they were planned (i.e., recent treatments weigh more than older ones).

We have not clinically validated the proposed nomograms, since on the one hand the results observed in this setting were already highly accurate (range residual spherical equivalent between − 1.12 D and + 0.75 D, and residual astigmatism below 0.75 D), and on the other hand no patient requested an enhancement.

However, we have virtually validated the different nomograms by assuming that the adjustment in the plan transfers linearly to the achieved change (Table 3). All proposals suggested minor improvements versus the actual observed outcomes, but there were no clinically relevant differences among the different nomograms.

**Table 3:** Comparison of the virtual refractive outcomes for all nomograms

	Post-op SEq (D) (Mean ± SD) (Range)	Post-op Cyl (D) (Mean ± SD) (Range)	*p* value compared to actual outcomes
Actual outcomes	−0.08 ± 0.36 − 1.12 to + 0.75	0.16 ± 0.21 0.00 to 0.75	–
Nomogram 1: Sph + negCyl	0.00 ± 0.35 − 1.20 to + 0.91	0.12 ± 0.17 0.00 to 0.75	< 0.0001
Nomogram 2: Sph + posCyl	0.00 ± 0.35 − 1.19 to + 0.95	0.12 ± 0.17 0.00 to 0.75	< 0.0001
Nomogram 3: SEq + Ast	0.00 ± 0.35 − 1.19 to + 0.93	0.12 ± 0.17 0.00 to 0.75	< 0.0001
Nomogram 4: Cyl_1_ + Cyl_2_	0.00 ± 0.35 − 1.20 to + 0.93	0.13 ± 0.17 0.00 to 0.78	< 0.0001
Nomogram 5: SEq + (C_+_,C_x_)	0.00 ± 0.35 − 1.19 to + 0.93	0.14 ± 0.16 0.02 to 0.79	< 0.0001
Nomogram 6: Cyclotorsion	0.00 ± 0.35 − 1.19 to + 0.93	0.14 ± 0.16 0.00 to 0.74	< 0.0001
Nomogram 7: SEq + C_+_ + C_x_	0.00 ± 0.35 − 1.20 to + 0.91	0.19 ± 0.17 0.05 to 0.80	< 0.0005

*Ast* = astigmatism; *C*_*+*_ = cardinal astigmatism; C_x_ = oblique astigmatism; *negCyl* = negative cylinder; *posCyl* = positive cylinder; *SEq* = spherical equivalent; *Sph* = sphere

One fact we must consider is that there are a number of sources of uncertainty (due to fluctuations in accommodation, uncertainty in point of final refraction, errors in working distance, uncertainties in the power of the trial lenses, errors in trial lens vertex distance) in the quoted values for manifest refraction [47]. Some of these can be analysed statistically, using the International Organization for Standardization (ISO) guidelines and can be used to estimate an uncertainty in the final recorded values. These uncertainties should be regarded as minimal because other factors such as unwanted accommodation are involved, which will affect the final outcome. The analysis showed that the uncertainty, which provides a 95% confidence level, would then be 0.6 D in refractive measurement, which is much larger than the 0.25 D steps in which manifest refraction used to be measured.

We based our analysis on simple power vectors [48] but not more comprehensive power matrices [49] that represent dioptric power in its full character because power vectors (with three components) are useful for adding, subtracting, and averaging powers of thin lens systems.

## Conclusion

The use of linear regression analysis to derive percentage and bias adjustments to attempted sphere and cylinder in laser vision correction has been well described in the past. Our analysis further suggests that for all the tested nomograms, minor improvements could be anticipated versus the actual observed outcomes, however without clinically relevant differences among them. The minimal uncertainties in determining the manifest refraction (~ 0.6 D) are the major limitation to improving the accuracy of refractive surgery nomograms; hence, more emphasis shall be placed on the estimation of manifest refraction with higher precision, for improving the treatment planning and accuracy in postoperative outcomes.

## References

[1] Yang SH, Van Gelder RN, Pepose JS (1998). Neural network computer program to determine photorefractive keratectomy nomograms. J Cataract Refract Surg.

[2] Feltham MH, Wolfe RJ (2000). Some variables to consider to avoid the need for LASIK surgical enhancements. Clin Exp Optom..

[3] Moniz N, Fernandes ST (2002). Nomogram for treatment of astigmatism with laser in situ keratomileusis. J Refract Surg.

[4] Alpins NA, Goggin M (2004). Practical astigmatism analysis for refractive outcomes in cataract and refractive surgery. Surv Ophthalmol.

[5] Gailitis RP (2005). Comparison of LASIK outcomes with the Alcon LADARVision4000 and the VISX STAR S2 excimer lasers using optimized nomograms. J Refract Surg.

[6] Lapid-Gortzak R, van der Linden JW, van der Meulen IJ, Nieuwendaal CP (2008). Advanced personalized nomogram for myopic laser surgery: first 100 eyes. J Cataract Refract Surg.

[7] Arnalich-Montiel F, Wilson CM, Morton SJ, Allan BD (2009). Back-calculation to model strategies for pretreatment adjustment of the ablation sphere in myopic wavefront laser in situ keratomileusis. J Cataract Refract Surg.

[8] Mattioli R, Tripoli NK (1997). Corneal geometry reconstruction with the Keratron videokeratographer. Optom Vis Sci.

[9] Salmon TO. Corneal contribution to the wavefront aberration of the eye. PhD Dissertation. 1999.

[10] Mrochen M, Jankov M, Bueeler M, Seiler T (2003). Correlation between corneal and total wavefront aberrations in myopic eyes. J Refract Surg.

[11] Mrochen M, Büeler M (2008). Aspheric optics: physical fundamentals. Ophthalmologe.

[12] Munnerlyn CR, Koons SJ, Marshall J (1988). Photorefractive keratectomy: a technique for laser refractive surgery. J Cataract Refract Surg.

[13] Yoon G, MacRae S, Williams DR, Cox IG (2005). Causes of spherical aberration induced by laser refractive surgery. J Cataract Refract Surg.

[14] Hersh PS, Fry K, Blaker JW (2003). Spherical aberration after laser in situ keratomileusis and photorefractive keratectomy. Clinical results and theoretical models of etiology. J Cataract Refract Surg.

[15] Calossi A (2007). Corneal asphericity and spherical aberration. J Refract Surg.

[16] Arba-Mosquera S, de Ortueta D (2008). Geometrical analysis of the loss of ablation efficiency at non-normal incidence. Opt Express.

[17] Bende T, Seiler T, Wollensak J. Side effects in excimer corneal surgery. Corneal thermal gradients. Graefe Arch Ophthalmol. 1988;226:277–80. 10.1007/BF02181196 3402751

[18] Huang D, Arif M (2002). Spot size and quality of scanning laser correction of higher-order wavefront aberrations. J Cataract Refract Surg.

[19] Guirao A, Williams DR, MacRae SM (2003). Effect of beam size on the expected benefit of customized laser refractive surgery. J Refract Surg..

[20] Arba-Mosquera S, Merayo-Lloves J, de Ortueta D (2008). Clinical effects of pure cyclotorsional errors during refractive surgery. Invest Ophthalmol Vis Sci.

[21] Bueeler M, Mrochen M (2005). Simulation of eye-tracker latency, spot size, and ablation pulse depth on the correction of higher order wavefront aberrations with scanning spot laser systems. J Refract Surg.

[22] Wirbelauer C, Aurich H, Pham DT (2007). Online optical coherence pachymetry to evaluate intraoperative ablation parameters in LASIK. Graefes Arch Clin Exp Ophthalmol.

[23] Camellin M, Arba Mosquera S. Aspheric Optical Zones: The Effective Optical Zone with the SCHWIND AMARIS. J Refract Surg. 2011;27:135–46. 10.3928/1081597X-20100428-03 20481411

[24] Steinert RF, Fynn-Thompson N (2008). Relationship between preoperative aberrations and postoperative refractive error in enhancement of previous laser in situ keratomileusis with the LADARVision system. J Cataract Refract Surg.

[25] Chen L, Artal P, Gutierrez D, Williams DR (2007). Neural compensation for the best aberration correction. J Vis.

[26] Marcos S, Cano D, Barbero S. Increase in corneal asphericity after standard laser in situ keratomileusis for myopia is not inherent to the Munnerlyn algorithm. J Refract Surg. 2003;19:S592–6. 10.3928/1081-597X-20030901-17 14518750

[27] Arbelaez MC, Vidal C, Arba-Mosquera S. Bilateral Symmetry before and Six Months after Aberration-Free™ Correction with the SCHWIND AMARIS TotalTech Laser: Clinical Outcomes. J Optom. 2010;3:20–8.

[28] De Ortueta D, Haecker C (2008). Laser in situ keratomileusis for mixed astigmatism using a modified formula for bitoric ablation. Eur J Ophthalmol.

[29] Cheng X, Bradley A, Thibos LN (2004). Predicting subjective judgment of best focus with objective image quality metrics. J Vis.

[30] Marsack JD, Thibos LN, Applegate RA (2004). Metrics of optical quality derived from wave aberrations predict visual performance. J Vis.

[31] Watson AB, Ahumada AJ Jr. Predicting visual acuity from wavefront aberrations. J Vis. 2008;8(4):17.1–19. 10.1167/8.4.17 18484856

[32] Subbaram MV, MacRae SM (2007). Customized LASIK treatment for myopia based on preoperative manifest refraction and higher order aberrometry: the Rochester nomogram. J Refract Surg.

[33] Artal P, Chen L, Fernández EJ, Singer B, Manzanera S, Williams DR (2004). Neural compensation for the eye's optical aberrations. J Vis.

[34] Mrochen M, Seiler T (2001). Influence of corneal curvature on calculation of ablation patterns used in photorefractive laser surgery. J Refract Surg.

[35] Jiménez JR, Anera RG, Jiménez del Barco L, Hita E (2002). Effect on laser-ablation algorithms of reflection losses and nonnormal incidence on the anterior cornea. Appl Phys Lett.

[36] Jiménez JR, Anera RG, Jiménez del Barco L, Hita E, Pérez-Ocón F. Correlation factor for ablation algorithms used in corneal refractive surgery with gaussian-profile beams. Opt Express. 2005;13:336–43. 10.1364/opex.13.000336 19488358

[37] Dorronsoro C, Cano D, Merayo-Lloves J, Marcos S (2006). Experiments on PMMA models to predict the impact of corneal refractive surgery on corneal shape. Opt Express.

[38] Dorronsoro C, Marcos S (2007). Experiments on PMMA model to predict the impact of corneal refractive surgery on corneal shape: reply. Opt Express.

[39] Kwon Y, Choi M, Bott S (2008). Impact of ablation efficiency reduction on post-surgery corneal asphericity: simulation of the laser refractive surgery with a flying spot laser beam. Opt Express.

[40] Kwon Y, Bott S (2009). Postsurgery corneal asphericity and spherical aberration due to ablation efficiency reduction and corneal remodelling in refractive surgeries. Eye (Lond).

[41] Ditzen K, Handzel A, Pieger S (1999). Laser in situ keratomileusis nomogram development. J Refract Surg.

[42] Sheludchenko VM, Fadeykina T (2001). Comparative results between standard and bitoric nomograms for astigmatism correction. J Refract Surg.

[43] Anderson NJ, Hardten DR, Davis EA, Schneider TL, Samuelson TW, Lindstrom RL (2003). Nomogram considerations with the Technolas 217A for treatment of myopia. J Refract Surg.

[44] Caster AI, Hoff JL, Ruiz R (2004). Nomogram adjustment of laser in situ keratomileusis for myopia and myopic astigmatism with the Alcon LADARVision system. J Refract Surg.

[45] Zaldivar R, Oscherow S, Bains HS (2005). Five techniques for improving outcomes of hyperopic LASIK. J Refract Surg.

[46] Mrochen M, Hafezi F, Iseli HP, Löffler J, Seiler T (2006). Nomograms for the improvement of refractive outcomes. Ophthalmologe.

[47] Smith G (2006). Refraction and visual acuity measurements: what are their measurement uncertainties?. Clin Exp Optom.

[48] Thibos LN, Horner D (2001). Power vector analysis of the optical outcome of refractive surgery. J Cataract Refract Surg.

[49] Harris WF (2007). Power vectors versus power matrices, and the mathematical nature of dioptric power. Optom Vis Sci.

